# Editorial: Reperfusion Therapy for Acute Ischemic Stroke

**DOI:** 10.3389/fneur.2019.01139

**Published:** 2019-10-29

**Authors:** Nishant K. Mishra, Bruce C. V. Campbell

**Affiliations:** ^1^Department of Neurology, Icahn School of Medicine at Mount Sinai, New York, NY, United States; ^2^Department of Medicine and Neurology, Melbourne Brain Centre at the Royal Melbourne Hospital, University of Melbourne, Parkville, VIC, Australia

**Keywords:** stroke, mismatch, endovascular, imaging, alteplase

Endovascular thrombectomy to recanalize large vessel occlusions in ischemic stroke patients is now proven to improve clinical outcomes in randomized trials (1–6). This resulted in significant excitement in the field because, until these trials, intravenous (IV) alteplase was the only proven therapy available to treat these patients (7–9). Also a little prior to the positive endovascular trials, the field was subdued because the major promising endovascular trials, IMS III and MR RESCUE, had failed to show improved outcomes from endovascular therapy (10, 11). These trials were believed to have failed because of the long delay between symptom onset and treatment, inadequate patient selection, lower recanalization rates, and the use of older generation devices. Stroke centers across the globe are now incorporating lessons learnt from the recent positive trials (1–6) into restructuring the acute stroke pathway to expeditiously transfer eligible patients to the neurointerventionist (12). We invited manuscripts (Drozdowska et al.; Etherton et al.; Fabian et al.; Fandler et al.; McKinley et al.; Taylor-Rowanet al.; van de Graaf et al.; Wouters et al.; Zhou et al.) to evaluate the current state of knowledge about reperfusion therapy with the goal of educating our readership about the patient selection paradigms, challenges in clinical trial design, and identifying future directions.

We now know that the benefit of endovascular recanalization in ischemic stroke patients with a target perfusion mismatch profile is independent of the time since symptom onset (13–16). However, in 2010, a meta-analysis of trials that included patients using perfusion mismatch for delayed window reperfusion therapy using desmoteplase and alteplase failed to demonstrate utility of the mismatch paradigm (17). An important limitation noted was that the mismatch criteria used in these studies used visual assessment or lenient thresholds (time-to-maximum [Tmax] > 2 s) for the perfusion lesion which included mildly hypoperfused tissue not at risk of infarction (benign oligemia) (17). Subsequent positive studies have used a Tmax > 6 s delay threshold (13–15, 17). Perfusion imaging data require complex analyses which can now be rapidly performed with automated post processing software e.g., RAPID (iSchemaView, Mountain View, CA) which was used in several pivotal trials to standardize recruitment criteria across different hardware platforms (18). Deconvolution algorithms rely on automated selection of an arterial input function which can lead to scan re-scan variation in Tmax and other perfusion parameters. There is therefore interest in using alternative measures like relative time-to-peak (rTTP) measures that normalize delay using a much larger area than the few voxels chosen for an arterial input function [Wouters et al.; (19)]. Wouters et al. contribute a manuscript to this collection reporting that a Tmax >6 s corresponds to rTTP >4.5 s and Tmax >10 s corresponds to rTTP >9.5 s. More importantly, the authors highlight the stability of rTTP in a scan-rescan scenario (Wouters et al.). McKinley et al. also acknowledge that deconvolution techniques are highly susceptible to noise and artifacts; they list measures to overcome these issues e.g., by adoption of a smoother residue function; and they report machine learning methodologies to identify the tissue-at-risk. The authors suggest that the best algorithms are those which are based on neural networks and random forests (McKinley et al.). Finally, Etherton et al. summarize the neuroimaging paradigms and the clinical trials that tested reperfusion therapy in stroke patients with unknown time of symptom onset. Perfusion mismatch, DWI-FLAIR mismatch, and other mismatch paradigms are discussed, and prognostic value of some of these measures discussed (Etherton et al.). The authors propose that more patients can be offered reperfusion therapy by refining the approach to identify additional populations of stroke patients with unknown onset who may benefit (Etherton et al.). The target of therapy—the tissue-at-risk (penumbra)—is better defined now and therefore there is greater opportunity to test preclinical research findings in the contemporary standard of care. To this end, we include Fabian et al. and Zhou et al. in this collection who report their data about methods to enhance ischemic preconditioning and to reduce oxidative stress respectively in acute stroke. Also, recanalization is not equivalent to reperfusion, and the goal should be to succeed with both (20). A large proportion of patients with successful recanalization do not show improved outcomes, possibly because of poor reperfusion, although the pre-treatment extent of injury is also a major contributor to poor outcome despite reperfusion. Periprocedural antithrombotic agents have a potential to improve reperfusion, but also have increased risk of intracranial bleeding. van de Graaf et al. conducted a systematic review to evaluate the use of periprocedural antithrombotic use in patients undergoing acute endovascular therapy. They found symptomatic ICH rate of 6–17% in patients with antiplatelet use and 5–12% in patients receiving heparin (van de Graaf et al.).

Endovascular stroke trials showed benefit in patients with proven large vessel occlusion and/or presence of mismatch. The investigators selected a population of patients with homogeneous clinical profile because the goal was to have a trial success (1, 5). In the real world, however, we often encounter patients who fall outside the randomized trial populations, and require individualized treatment decisions. Fandler et al. present one example as a case report highlighting excellent outcomes from endovascular therapy in a patient with ischemic stroke who had a stroke recurrence within 9 days of the first thrombectomy. The subsequent stroke occurred in exactly the same vascular territory, was associated with significant mismatch, and continued to show irregular shaped ulcerated plaque. Fandler et al. offered endovascular treatment with TICI 3 recanalization with excellent outcomes on follow up. She also received carotid thrombo-endarterectomy for the ulcerated plaque Fandler et al.. Patients treated off label are a highly heterogeneous group and therefore difficult to assess in randomized clinical trials. There is a need to collate these data in a registry setting.

Suboptimal trial design may well have been responsible for some of the neutral stroke trials reported in the last two decades. Drozdowska et al. and Taylor-Rowan et al. touch upon some of the relevant issues. Drozdowska et al. reviewed prognostic scales used in acute stroke and inform the readers about the validity of commonly used prognostic scores. Outcome prediction tools guide a stroke physician's discussion with patient's family in regard to possible outcomes. It also offers a clinical trialist a tool to stratify the patient population enrolled in studies to investigate a differential response to the treatment. Drozdowska et al., in their review, recommend the need for studies to investigate clinical usefulness in existing scales. Outcome measures used in complex trials like stroke trials should be valid, reliable, and responsive endpoint; and their analyses should be robust and acceptable to the regulatory bodies. In their review article, Taylor-Rowan et al. inform issues relevant to the selection of outcome measures and suggest robust analytics.

This Research Topic thus informs the current state of knowledge with respect to recanalization strategies and also stimulates readers to identify important research questions and tackle them through a robust research methodology.

## Author Contributions

All authors listed have made a substantial, direct and intellectual contribution to the work, and approved it for publication. Opinions are personal and do not reflect that of the regulatory bodies or the authors' employers.

### Conflict of Interest

The authors declare that the research was conducted in the absence of any commercial or financial relationships that could be construed as a potential conflict of interest.
